# Supplementary material for: Active site specificity profiling datasets of matrix metalloproteinases (MMPs) 1, 2, 3, 7, 8, 9, 12, 13 and 14
Source: Data Brief. 2016 Feb 22;7:299–310. doi: 10.1016/j.dib.2016.02.036 (PMC4777984; doi:10.1016/j.dib.2016.02.036)
Supplement: Supplementary file 10 — Supplementary material [file mmc10.zip › WebPICS_hMMP2_G_1%/P2prime.html]

 

PICS results


|  |  |
| --- | --- |
| **P2prime\_F**  15 in 246 sites   6.1 %    effects > 10 perc. pnts.  (vice-versa in brackets)  P1\_N: 17.4 (11.3) |  |
| **P2prime\_I**  35 in 246 sites   14.2 %    effects > 10 perc. pnts.  (vice-versa in brackets)  P2\_Q: 12.7 (24.7)   P1\_S: 16.4 (19.1)   P3prime\_N: 15.6 (30.2) |  |
| **P2prime\_K**  31 in 246 sites   12.6 %    effects > 10 perc. pnts.  (vice-versa in brackets)  P3\_V: 14.0 (15.0)   P3prime\_K: 12.1 (20.7) |  |
| **P2prime\_M**  7 in 246 sites   2.8 %    effects > 10 perc. pnts.  (vice-versa in brackets)  P1\_Q: 24.1 (15.4)   P1prime\_M: 24.5 (17.2) |  |
| **P2prime\_N**  10 in 246 sites   4.1 %    effects > 10 perc. pnts.  (vice-versa in brackets)  P3\_I: 66.6 (20.1)   P1\_G: 65.0 (17.5)   P3prime\_V: 79.0 (29.2) |  |
| **P2prime\_Q**  14 in 246 sites   5.7 %    effects > 10 perc. pnts.  (vice-versa in brackets)  P2\_Q: 14.1 (11.0)   P1\_N: 19.3 (11.7) |  |
| **P2prime\_T**  20 in 246 sites   8.1 %    effects > 10 perc. pnts.  (vice-versa in brackets)  P1prime\_C: 11.7 (29.4) |  |
| **P2prime\_Y**  8 in 246 sites   3.3 %    effects > 10 perc. pnts.  (vice-versa in brackets)  P3\_L: 39.4 (12.1)   P1prime\_W: 10.5 (16.7) |  |
